# The first discovery of severe fever with thrombocytopenia syndrome virus in Taiwan

**DOI:** 10.1080/22221751.2019.1710436

**Published:** 2020-01-10

**Authors:** Tsai-Lu Lin, Shan-Chia Ou, Ken Maeda, Hiroshi Shimoda, Jacky Peng-Wen Chan, Wu-Chun Tu, Wei-Li Hsu, Chi-Chung Chou

**Affiliations:** a College of Veterinary Medicine, National Chung Hsing University, Taichung, Taiwan; b National Institute of Infectious Disease, Tokyo, Japan; c Joint Faculty of Veterinary Medicine, Yamaguchi University, Yamaguchi, Japan; d Department of Entomology, National Chung Hsing University, Taichung, Taiwan

**Keywords:** severe fever with thrombocytopenia syndrome virus, first discovery in Taiwan, ticks, sheep, bovine, public health

## Abstract

Severe fever with thrombocytopenia syndrome (SFTS), an emerging tick-borne zoonosis, has been rapidly spread in many Asian counties since 2010, which raises the great concern in East Asia. Nevertheless, the infection status of SFTS in Taiwan remains unclear. To investigate the existence of SFTSV in Taiwan, a total of 151 serum samples collected from 31 sheep, 63 bovine and 57 dogs were enrolled this study. Furthermore, 360 adult female *Rhipicephalus microplus* were also included. One-step RT-nested PCR and IgG ELISA were conducted to test SFTSV specific RNA and antibodies, respectively. The result provided the first evidence of the existence of SFTSV RNA and antibodies in ruminants and ticks in Taiwan.

Severe fever with thrombocytopenia syndrome virus (SFTSV), a member of the family *Bunyaviridae*, genus *Phlebovirus*, is an emerging tick-borne zoonotic etiologic agent [1]. Clinical signs of SFTS in humans include high fever, vomiting, diarrhoea, thrombocytopenia, leukopenia, and multiple organ failure with the mortality rates of the disease ranging from 6% to as high as 30% [2]. For the transmission of the virus, ticks, *Haemaphysalis longicornis* and *Rhipicephalus microplus* are suspected to be the first and second main potential vectors [3]*.* Moreover, SFTSV is well-known for the wide range of host species, including sheep, cattle, dogs, pigs, chicken and other wildlife [4]; nonetheless, herbivorous animals were regarded as the amplifying host of SFTSV. However, until now, many questions remain unclear; in particular, the ecologic life cycle of SFTSV, the impact on animals, pathogenesis in various hosts, and its geographical distribution.

Since the first discovery of SFTSV in Hubei in China in 2010, the disease has rapidly spread to Fujian province which is only a strait away from Taiwan [1]. Later in 2012–2013, the first SFTS human case in Japan and South Korea were reported [5]. From 2013 to 2016, there were a total of 7419 confirmed human cases occurred in China with 355 deaths [1]. In 2019, SFTSV RNA was detected from two human serum samples in Vietnam [6]. So far, the annual cases were continually increasing, which raises a great public health concern in East Asia [1]. Due to the geographic proximity, and the intensive communication, i.e. flights and large cargo volume, between Taiwan and the endemic areas (China, Japan, and South Korea), it is worthy of investigation the status of SFTS in Taiwan.

As SFTS is a tick-borne infectious disease, a free-range farm in Nantou County was selected for this surveillance due to the high frequency of tick-infestation. In total, 151 sera including, 31 sheep, 63 bovine, and 57 canine samples from either companion (30) or shelter (27) dogs, were enrolled in this study. Moreover, 360 ticks were collected from bovine.

Initially, RT–PCR was performed to amplify the partial small (S) segment RNA (346 bp) of SFTSV [7]. Overall, SFTSV RNA was detected in 29% (9/31), 4.8% (3/63) of the sheep and bovine specimens, respectively, while it was not detected in dog samples (Table 1). The 360 ticks, all identified as female adult *R. microplus*, were pooled into 36 sample pools; among those, 9 (25%) showed positive results. Sequences of all the positive samples were validated by automated sequencing (Mission Biotech, Taiwan) and analysed using MEGA 7 [8]. The 5 distinct partial S sequences identified from sheep, bovine, and ticks indicated high similarity and were alternately present in the same clade (Figure 1), suggesting a close phylogenetic relationship and a likelihood of circulating between the ruminants and the ticks. Of note, the S gene amplified from two pooled tick samples harboured 12-nucleotide deletion (supplementary Figure S1). Subsequently, an attempt was made to identify the sequence of the full length S gene segment from one of the sheep samples with the highest level of viral RNA. Phylogenetic analysis of the full length S segment indicated our SFTSV was clustered into genotype C4, which is closely related to the Japanese strain (AB985572) and in the same clade which consists of strains isolated from China in majority (supplementary Figure S2).

**Figure 1.**
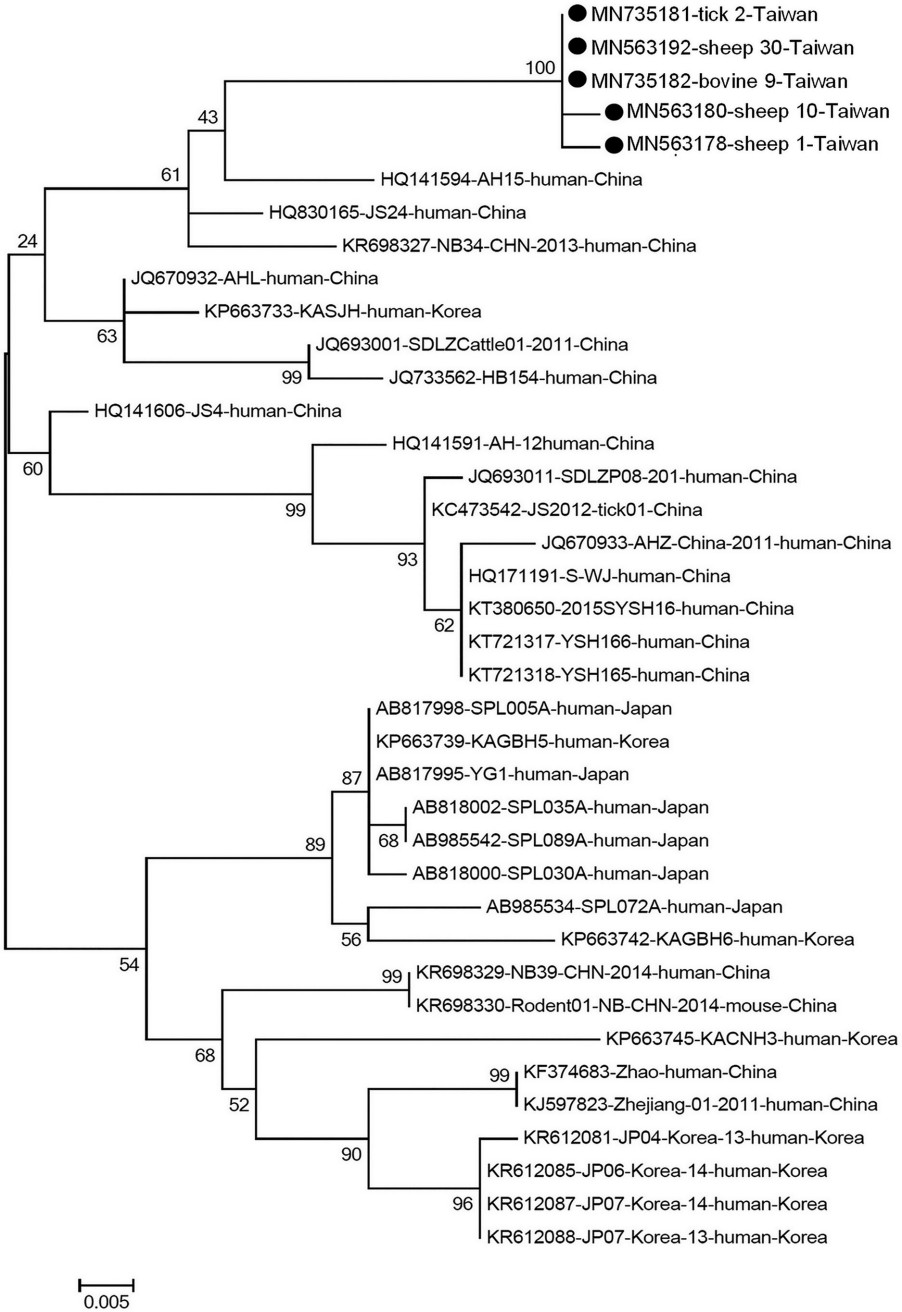
Phylogenetic analysis of the partial S segment of severe fever with thrombocytopenia syndrome virus identified in Taiwan. In total, of those 21 positive samples, 5 distinct sequences of partial S gene were identified from each species of animals in this study. The Nucleotide sequences of the local isolate indicated as a black circle. Other representative viral strains were presented with their accession numbers and also the host and country of isolation. The evolutionary history was inferred using the maximum-likelihood method, based on the Kimura 2-parameter model (1000 bootstrap replicates). The percentage of trees in which associated taxa clustered is shown next to the branches. Scale bar indicates nucleotide substitutions per position.

**Table 1.** Percentage of sero-prevalence and RNA detection in animals in this study.

	Sheep (*n* = 31)	Bovine (*n* = 63)	Canine (*n* = 57)	Tick pools (*n* = 36)
RNA-positive (%)	29 (9/31)	4.8 (3/63)	0	25 (9/36)
Seropositive (%)	38.7 (12/31)	0	0	ND

Note: ND: not determined.

Moreover, seroprevalence was further conducted based on the detection of SFTSV-specific antibodies by ELISA [9]; in brief, the plate that was pre-coated with antigen, either SFTSV- or mock-infected Huh7 cell lysates, was reacted with 1:100 diluted serum samples followed by incubation with the HRP-conjugated protein A/G (serving as the secondary antibody). As summarized in Table 1, among the serum samples analysed, SFTSV-specific antibodies were presented in approximately 38.7% (12/31) in sheep, and of the 12 sheep samples with SFTSV antibody, four (33%) were also detected positively in PCR. Nevertheless, SFTSV antibody was not detected in both bovine and canine samples. It is worth noting that of 3 out of 63 bovine serum samples carry SFTSV-RNA without detectable antibody level. We suspected that it might be due to the higher sensitivity of nested PCR as compared with ELISA, the lower level of SFTSV antibody of bovine, or early disease onset.

Study herein demonstrated the presence of the RNA and antibodies of SFTSV in sheep and bovine, suggesting the existence of SFTSV in Taiwan. Of note, unlike that in humans, in this study, the animals detected SFTSV RNA-positive were without suspected clinical illness that is consistent with previous reports [10,11]. For the transmission route, Yun *et al*. have mentioned that migratory birds with ticks may serve as the long-distance carrier spreading SFTSV across borders, and Taiwan is on the path of the migratory route [12]. Despite the absence of the well-recognized vector, *Haemaphysalis longicornis*, the other two suspected vectors responsible for SFTSV transmission, namely *Amblyomma testudinarium* and *R. microplus* [13], are the common tick species in the field of Taiwan. As SFTSV RNA was readily detected in tick, *R. microplus*, it is possible that migratory birds with the virus containing-ticks may play an important role in the transmission of SFTSV to Taiwan.

This report reveals, for the first time, the presence of SFTSV in animals and ticks in Taiwan, indicating a broader global distribution of SFTS. Of note, while preparation of this manuscript, the Taiwan Centres for Disease Control (Taiwan CDC) announced the first human case of SFTS on 19 November 2019 (https://www.cdc.gov.tw/Bulletin). Since this patient did not travel abroad but with the history of mountain activities before disease onset, it is likely the source of the infection is present in Taiwan and that is in line with the findings herein. As the virus was readily detected in susceptible amplifying hosts and vectors of one free-range farm; in order to control SFTSV, surveillance of the SFTSV prevalence should be implemented to a nationwide level and extended to a wider host range including the possible vector species in Taiwan.

## Supplementary Material

Supplemental Material
