# Supplementary material for: The first discovery of severe fever with thrombocytopenia syndrome virus in Taiwan
Source: Emerg Microbes Infect. 2020 Jan 10;9(1):148–51. doi: 10.1080/22221751.2019.1710436 (PMC6968498; doi:10.1080/22221751.2019.1710436)
Supplement: Supplemental Material [file TEMI_A_1710436_SM9054.docx]

**Supplementary Figure**


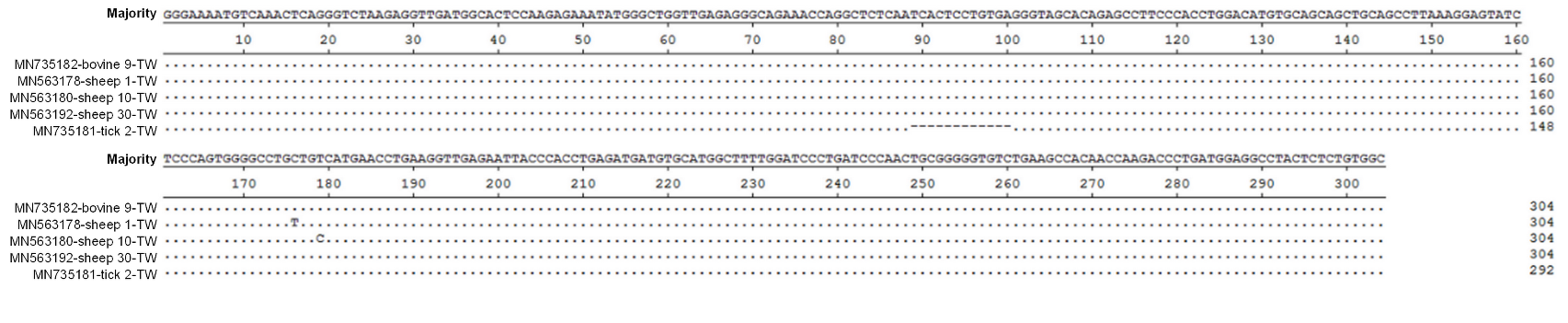


**Supplementary Figure S1.** Alignment of the 304 bp of the partial SFTSV S segment in mammals and tick samples.

Of those 21 positive samples amplified from ruminants and ticks, all sequences shared 95-100% of similarity. Samples with distinct sequences of each animal species were aligned. Sequence variations including single point substitutions and 12-nucleodite deletion (indicated as dash lines) were noticed.

**
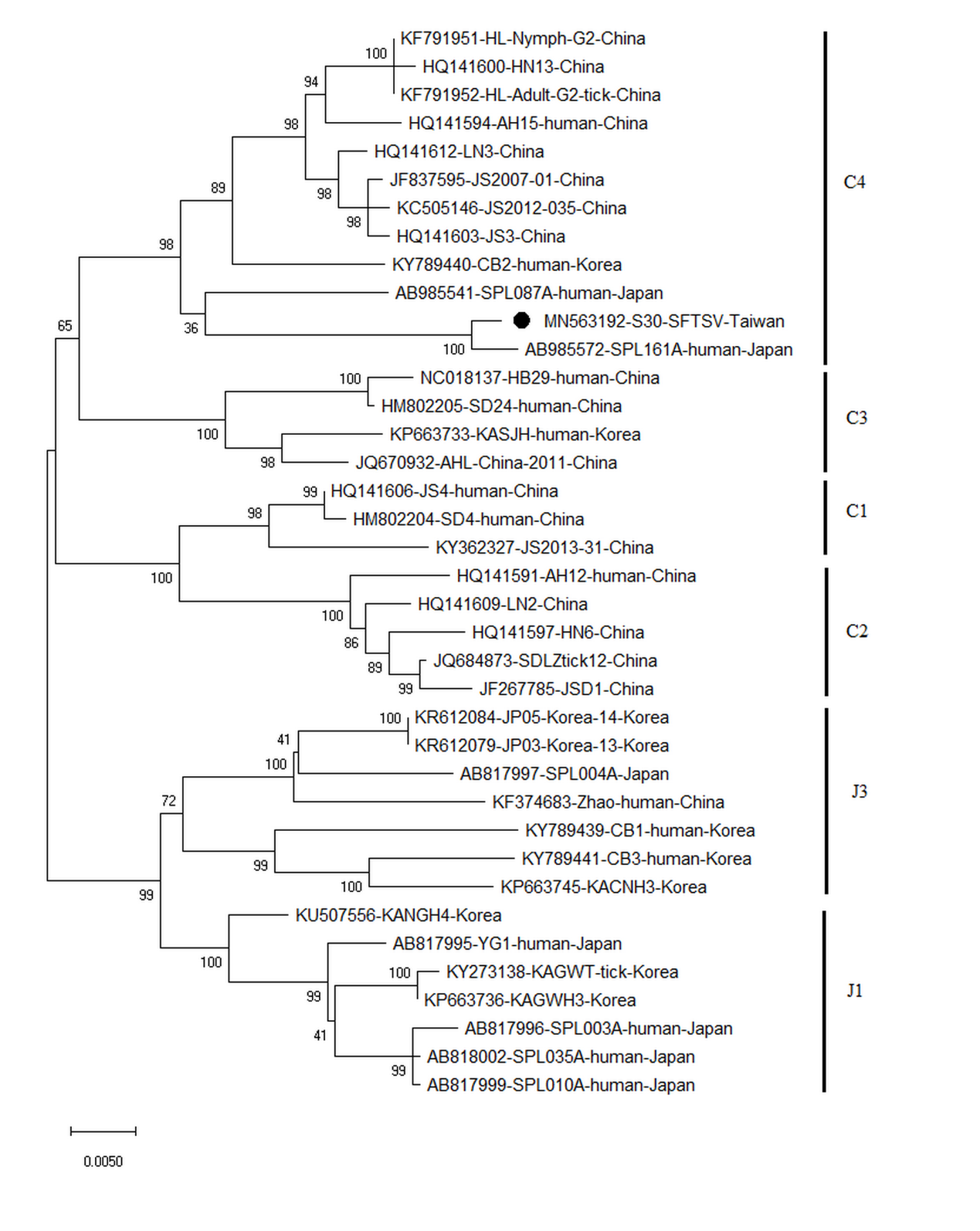
**

**Supplementary Figure S2.** Phylogenetic analysis of the full length S segment of severe fever with thrombocytopenia syndrome virus identified in Taiwan.

The Nucleotide sequences of the S segment of our local isolate (accession number MN563192) was indicated as a black circle. Other representative viral strains indicating the genotypes of SFTSV in the tree were shown by their accession number with the country of origin. The evolutionary history was inferred using the maximum-likelihood method, based on the Kimura 2-parameter model (1,000 bootstrap replicates). The designation of major viral genotype was referred to as the previous study [1]. The percentage of trees in which associated taxa clustered is shown next to the branches. Scale bar indicates nucleotide substitutions per position.

References

1. Yoshikawa T, Shimojima M, Fukushi S, et al. Phylogenetic and Geographic Relationships of Severe Fever With Thrombocytopenia Syndrome Virus in China, South Korea, and Japan. The Journal of infectious diseases. 2015 Sep 15;212(6):889-98. doi: 10.1093/infdis/jiv144. PubMed PMID: 25762790.
